# Using Real-World Data on Depression from EHR-based Research Networks: A Scoping Review

**DOI:** 10.21203/rs.3.rs-7272352/v1

**Published:** 2025-08-05

**Authors:** Yujia Sun, Dmitry Scherbakov, Gaylen Fronk, Leigh Ridings, Alexander V. Alekseyenko, Leslie A. Lenert

**Keywords:** depression, electronic health records, EHR, research networks, scoping review

## Abstract

**Introduction:**

Depression poses a significant global public health challenge, necessitating innovative research to understand its epidemiology and management. Electronic health record (EHR) research networks offer a powerful tool to study depression at scale, yet remain underutilized. This scoping review summarizes the extent of depression research ongoing in EHR networks.

**Methods:**

Following the Arksey and O’Malley framework and PRISMA guidelines, we searched PubMed, Scopus, EBSCOHost, and Google Scholar in September 2024, identifying 166 studies from 1211 records. Included studies used EHR networks like TriNetX, All of Us, and the Million Veteran Program (MVP) to investigate depression, defined broadly to include various depressive disorders. Covidence with custom large language model (LLM) plugin was used to aid screening and extraction processes.

**Results:**

Depression research in EHR networks is limited, with TriNetX (36 studies) and All of Us (24 studies) the most utilized platforms. Populations studied were predominantly from the United States (125 studies), followed by Canada (5) and European countries (15 combined). Common predictors analyzed included age (58 studies), gender/sex (56 studies), and race/ethnicity (45 studies).

**Conclusion:**

EHR networks hold vast real-world data for advancing depression research, but underutilization highlights the need for better accessibility to enhance future studies.

## INTRODUCTION

Depression remains a major public health challenge worldwide, with significant social, economic, and healthcare burdens^1^. Epidemiological studies have identified numerous risk factors for depression, including biological, socio-demographic, and environmental factors, which vary across populations^2^. The ability to leverage large-scale electronic health record (EHR) data for depression research presents a unique opportunity to advance understanding of its prevalence, risk factors, treatment patterns, and outcomes^3^. EHR research networks have become invaluable tools for aggregating and analyzing realworld clinical data, enabling large, diverse population-based studies that would otherwise be unfeasible^4^. These networks not only provide extensive data on patient demographics, diagnoses, and treatments, but also facilitate longitudinal tracking of patient outcomes, allowing for more nuanced insights into the course of depression across different population groups.

EHR data are collected from everyday clinical settings. These real world data enable creating cohorts numbering in the tens of thousands to millions, across broad health systems, capturing patients otherwise underrepresented in clinical trials (e.g., elderly, highly comorbid, minority groups), thus supporting greater generalizability of findings^5^. The longitudinal nature of EHR data enables sophisticated and causal mapping of disease course, including onset, recurrence, treatment changes (e.g., pharmacotherapy switching, augmentation), and time to key events such as crisis or treatment resistance—features largely inaccessible in traditional datasets^6^. Several EHR research networks, including TriNetX, Million Veterans Program (MVP), ENACT, PCORNet, AllofUs, Epic Cosmos, have emerged as central platforms in the study of various health outcomes, including depression.

The primary research question guiding this scoping review is: What is the scope of depression research projects using EHR research networks and databases? We followed scoping review recommendations outlined in Arksey and O’Malley^7^ to explore the scope of depression research using these EHR networks and databases, providing a comprehensive overview of the types of studies conducted, their objectives, and key findings. The secondary research goal was to identify the phenotyping methods used for depression diagnoses in EHR research networks and the socio-demographic variables employed during statistical modeling. This study aims to fill critical gaps in understanding how these large datasets contribute to the growing field of depression research, while also evaluating the consistency of findings across different networks.

## MATERIALS AND METHODS

This review follows the checklist for scoping review outlined by PRISMA^8^. Citation search was conducted across four citation databases in September 2024: abstract/title search in PubMed, Scopus, EBSCOHost (including CINAHL Complete, APA PsycInfo, APA PsycArticles), and full-text search in Google Scholar (limited to the first 40 results). The search strategy included the following terms:

Inclusion and exclusion criteria were developed to assess the relevance of studies based on the abstract and full-text screening. Papers were included if they met the following criteria:

### Inclusion Criteria:

- The study used data from EHR research networks such as TriNetX, MVP, ENACT, PCORNet, AllofUS, Epic Cosmos, ODSI, National Clinical Cohort Collaborative (N3C), Datavant Switchboard, Cerner Real-World Data, Optum EHR, Truveta, or any other health records research network.
- The paper discussed depression, defined broadly to include mild depression, dysthymia, major depressive disorder, or other depressive disorders (excluding bipolar disorder).
- Full-text article with sufficient detail was available.

### Exclusion Criteria:

- Papers not using a research network but instead relying on localized EHR databases, such as hospital-specific records.
- Studies that did not report data on depression.
- Review papers, book chapters, and other non-original research articles (e.g., systematic reviews, scoping reviews, literature reviews).
- Papers with abstracts that were too brief or lacked sufficient detail to evaluate the study.

For each included study, the following data were extracted:

- **EHR Network Used**: Identifying the specific research network or database used for the depression study.
- **Depression Diagnosis**: The definition of depression used, including any ICD, DSM-5, or other coding systems applied to phenotype depression patients.
- **Validation Process**: Whether any validation steps, such as manual chart reviews or natural language processing algorithms, were used to confirm depression diagnoses.
- **Symptoms Measurement**: Whether depression symptoms were measured using validated tools such as CES-D, PHQ-9, or BDI.
- **Socio-demographic Breakdown**: Reporting of depression diagnoses or symptoms by gender, age group, income level, and other demographic variables (e.g., rural vs. urban, marital status, race/ethnicity).
- **Geography of Study Population**: The geographical scope of the population covered in the study.
- **Study Type and Statistical Models**: Whether depression was the outcome variable in the study, and what predictors (e.g., health conditions, demographic factors) were used in statistical or machine learning models, if any).

All citations were then uploaded to Covidence. Covidence was used as a review protocol to track the progress of the study. The screening and extraction process was conducted by two human reviewers (DS and GF) with the help of a large language model (LLM) Covidence plugin developed by the Medical University of South Carolina. The plugin and the process is detailed in the separate paper.^9^ All outputs of LLMs were benchmarked by a human reviewer (GS). The benchmarks for LLM during screening are provided in Supplementary Tables S1-S3, while the LLM prompts used are provided in Supplementary Table S4.

The extraction data and results were synthesized by JS. Grok LLM was used to assist in drafting of the review and in narrative synthesis of the Results subsections using Supplementary File S5 information. Python was used to produce frequency statistics and figures.

## RESULTS

### Study selection process

**Figure 1:**
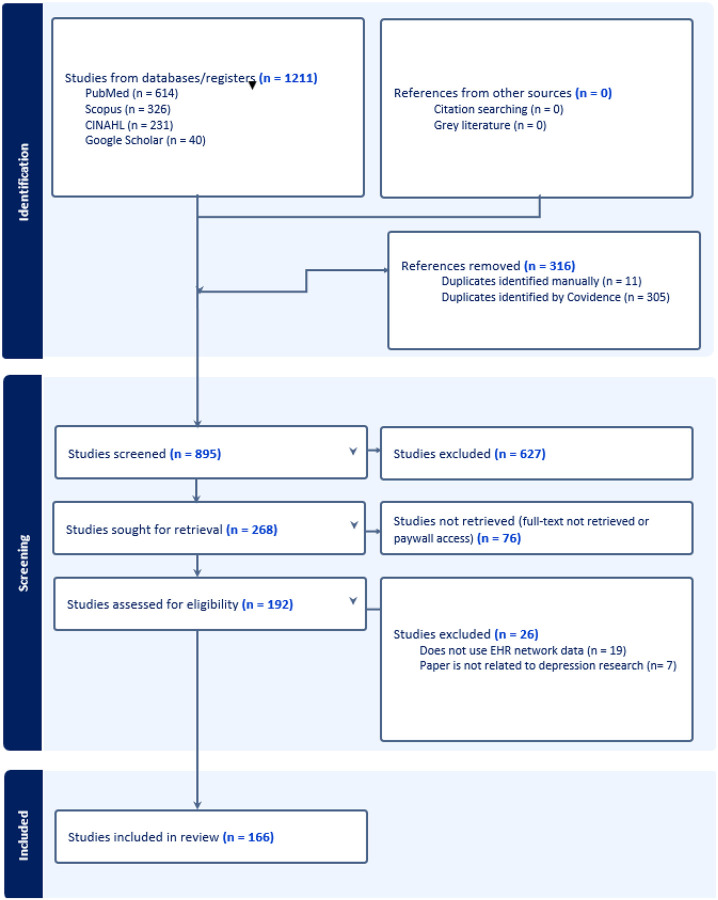
PRISMA Flow chart

The study selection process is summarized in the PRISMA flow diagram (Fig. 1). The initial search across databases and registers yielded 1211 studies. After removing 316 duplicates (11 identified manually and 305 by Covidence), 895 studies remained for screening. During the screening phase, 627 studies were excluded according to exclusion criteria. Of the remaining 268 studies sought for retrieval, 76 could not be retrieved due to full-text unavailability or paywall restrictions. This left 192 studies assessed for eligibility. Finally, 26 studies were excluded because they either did not use EHR network data (19 studies) or were not related to depression research (7 studies). Ultimately, 166 studies were included in this systematic review.

### Summary of included studies

Table 2 provides a summary table of reviewed studies with networks which were mentioned in more than one study included. All networks and the full details of extracted studies are provided in Supplementary File S5. The top 3 research networks were: TriNetX, featured in 36 studies; The All of Us research program, featured in 24 studies; and The MVP (Million Veteran Program) which was utilized in 19 studies.

**Figure 3.**
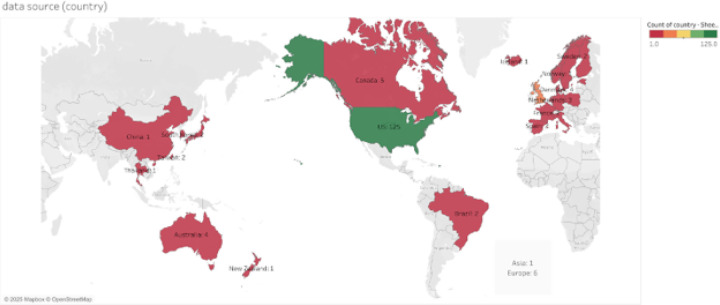
Geography of populations studied in research networks. The legend indicates a color gradient from red (1) to green (125), representing the count of studies per country, visually emphasizing the dominance of US-based data sources

Figure 3 illustrates the geographic distribution of the data sources utilized in the included studies. The map highlights a significant concentration of research originating from the United States, which contributed data for 125 studies.

**Figure 4.**
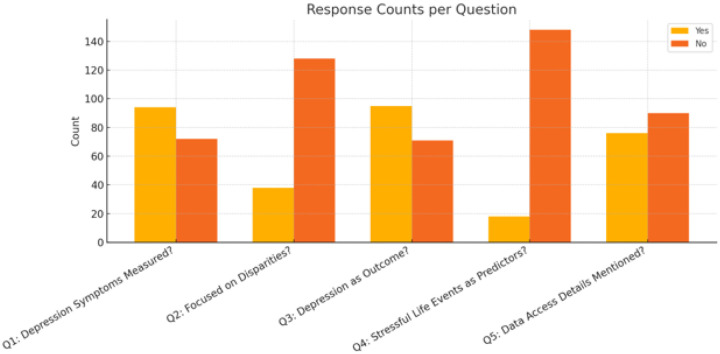
Primary characteristics of Included Studies

Figure 4 summarizes the studies by specific criteria related to key study characteristics. Most studies (n = 95) used depression symptom measures and the same amount featured depression as the primary outcome. A smaller amount of studies were dedicated to depression disparities and stressful life events. The majority of studies (n = 95) didn’t provide details on how EHR network data could be accessed.

### Depression as a Primary Outcome

Numerous studies (n = 95) focus on depression as a primary outcome, leveraging EHR data to estimate prevalence, incidence, or severity across diverse populations and contexts. These studies often utilize large-scale EHR databases to analyze depression in specific medical conditions, such as chronic kidney disease, cancer, or neurological disorders.^82–84^ Others employ EHR data to assess depression during public health events like the COVID-19 pandemic, examining its impact on healthcare workers, young adults, or those with chronic conditions.^55,64,85,86^ Research also uses EHRs to explore genetic and environmental factors, such as polygenic risk scores or social determinants like discrimination and social support, in relation to depression.^48,50,57,58,62,87^ Additionally, studies of specific populations, such as veterans or individuals with dermatologic or autoimmune conditions, rely on EHR data to characterize depression outcomes.^61,63,88–90^ EHR-based predictive models and intervention evaluations further enhance the understanding of depression treatment outcomes.^68,91–94^

### Depression as a Comorbid or Secondary Outcome

Another group of studies examines depression as a secondary or comorbid outcome, often using EHR data to explore its interactions with other conditions or interventions. These studies analyze EHRs to assess depression alongside physical or psychiatric conditions, such as cardiovascular diseases, diabetes, or substance use disorders.^75,95,96^ Research also leverages EHR data to investigate depression in the context of surgical or medical interventions, including bariatric surgery, lumbar fusion, or gender-affirming procedures.^28,39,97^ Additionally, studies use EHRs to evaluate depression alongside other mental health outcomes, like anxiety or PTSD, in populations with chronic illnesses, trauma, or infectious diseases.^9,20,33,34,40,98^ EHR data are particularly valuable in studying depression in specific demographics, such as veterans or individuals with HIV, to understand its interplay with other health outcomes.^30,78,99^

### Depression as an Independent Variable or Risk Factor

Several studies treat depression as an independent variable, using EHR data to explore its influence on other health outcomes. These include investigations into how depression affects recovery from conditions like COVID-19, stroke, or traumatic brain injury, with EHRs providing detailed clinical and demographic data.^26,38,100^ Others use EHRs to examine depression’s impact on treatment outcomes, such as weight loss in bariatric surgery or survival in cancer patients.^25,97^ Research also relies on EHR data to assess depression as a risk factor for outcomes like suicidality, substance use disorders, or cardiovascular events.^15,23,23,81^ Additionally, EHRs facilitate studies on how depression mediates relationships between conditions like dermatologic diseases or chronic illnesses and outcomes such as alcohol use or quality of life.^101,102^

### Mental Health Outcomes Beyond Depression

Some studies focus on mental health outcomes like suicidality, anxiety, or PTSD, where depression is a covariate or related condition, often utilizing EHR data for comprehensive analyses. These include research on suicide risk in populations with specific medical histories, such as traumatic brain injury or gender-affirming procedures, with EHRs enabling detailed risk profiling.^10,12,13,23,74^ Others explore genetic architectures of psychiatric disorders like PTSD or schizophrenia, using EHRs to link clinical data with genetic findings and note overlaps with depression.^77,78,80,103^ Studies also leverage EHRs to investigate mental health outcomes in chronic conditions like HIV or kidney disease, where depression is one of several comorbidities.^96,104,105^

### Health System and Treatment Patterns

A distinct area of research uses EHR data to analyze health system dynamics, treatment patterns, and interventions for mental health, including depression. These studies examine antidepressant prescribing patterns, the impact of health policies, or the effectiveness of EHR-based screening tools in primary care settings.^106–109^ Others utilize EHRs to evaluate the implementation of evidence-based practices or collaborative care models to improve mental health outcomes, including depression.^110–112^ EHR data are critical in studies enhancing diagnosis, personalizing treatment, or predicting mental health outcomes through advanced analytics.^6,113,114^

### Depression Disparities

Several studies leverage EHR data to investigate disparities in depression prevalence, diagnosis, or treatment across demographic groups, emphasizing racial, ethnic, socioeconomic, or gender-based differences. These studies often use EHRs to identify variations in depression outcomes among underrepresented groups, such as sexual and gender minorities, veterans, or individuals with specific medical conditions.^44,65,115,116^ Research also examines how social determinants, such as discrimination or socioeconomic status, contribute to disparities in depression, with EHRs providing granular data on patient demographics and clinical encounters.^48,62^ Additionally, studies explore disparities in antidepressant prescribing or access to mental health care, particularly in vulnerable populations like veterans or those with chronic illnesses, using EHRs to track treatment patterns and outcomes.^46,107^

### Utilization of Stressful Life Events

A subset of studies uses EHR data to explore the role of stressful life events as risk factors or predictors of depression and related mental health outcomes. These studies often integrate EHRs with genetic or environmental data to examine how stressful events, such as trauma or significant life changes, interact with clinical or genetic profiles to influence depression.^87,103^ Research also leverages EHRs to assess the impact of stressful events during public health crises, like the COVID-19 pandemic, on depression and other mental health conditions, capturing detailed patient histories and social determinants.^48,57,58^ Additionally, studies use EHR data to investigate how stressful life events, such as those related to medical diagnoses or social stressors, contribute to mental health outcomes in specific populations, including veterans or individuals with chronic conditions.^74,78^

### Social and Environmental Influences

Several studies investigate social and environmental factors influencing mental health, often using EHR data to assess depression as a key outcome. These include research on the impact of discrimination, social support, or socioeconomic disparities during crises like the COVID-19 pandemic, with EHRs providing granular demographic and clinical data.^48,57,58,115^ Others use EHRs to explore lifestyle factors, such as physical activity or maternal health, and their associations with depression or related outcomes.^16,117,118^ EHR data also support research on social determinants like stigma or health disparities in underrepresented groups, enhancing the understanding of depression’s broader context.^62,99^

### Predictors used in the studies

**Figure 5.**
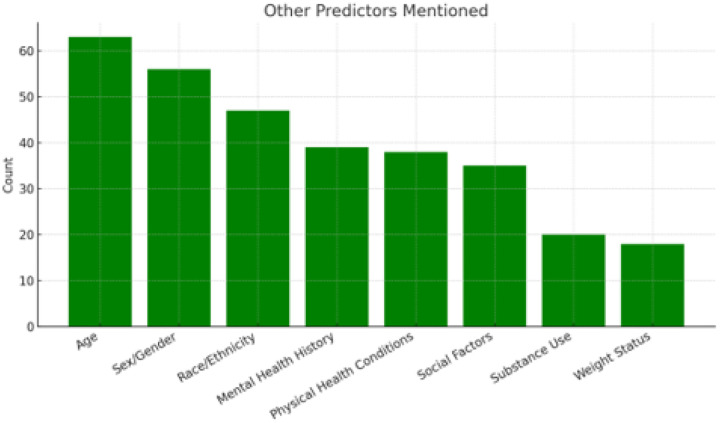
The frequency of different predictors analyzed in models with depression as an outcome

Figure 5 displays a bar chart illustrating the frequency of predictors—other than depression (if depression wasn’t the outcome it had to be a predictor for study to be reviewed) — that were most consistently mentioned in the reviewed studies. “Age” was the most commonly reported predictor (63 studies), followed by “sex/gender” (56 studies) and “race/ethnicity” (47 studies), showing that demographic factors are some of the most frequently looked at in EHR-based depression research. “Mental health history” was considered in 39 studies and typically included variables such as prior depression diagnoses, anxiety disorders, and general psychiatric history. “Physical health conditions” (38 studies) encompassed comorbidities such as type 2 diabetes, hypertension, obesity, coronary artery disease (CAD), and chronic kidney disease. “Social factors” (35 studies) included marital or relationship status, socioeconomic status, and living arrangements, reflecting an effort to assess the social environment influencing mental health outcomes. “Substance use,” mentioned in 20 studies, referred to alcohol consumption, smoking, and drug misuse. “Weight status” (18 studies) typically involved body mass index (BMI) or clinical classifications of obesity.

### Depression phenotyping methods

**Figure 6.**
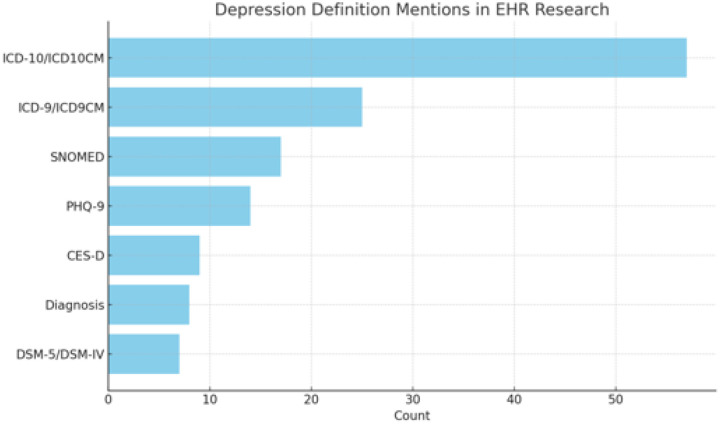
How depression phenotype is defined in the study

Figure 6 illustrates the frequency of various depression definition codes used in the study to identify the depression phenotype. In the reviewed studies, ICD-10/ICD-10-CM codes were the most frequently used definitions for depression (57 studies), with F32 (Depressive episode) and F33 (Recurrent depressive disorder), both cited 29 times. ICD-9/ICD-9-CM codes followed with 25 mentions, commonly including 296.2x, 296.3x, and 311, typically appearing in studies using older datasets. Among screening tools, the PHQ-9 (14 studies), with a cutoff score > 10 most often used (in 8 studies), followed by CES-D (9 studies) with varied thresholds. SNOMED was cited 17 times, with 35489007 (Depressive disorder) being the most frequent. DSM-5/DSM-IV were used in 7 studies, and general “diagnosis” terms were found in 8. Less frequently used definitions included tools like PHQ-2, HADS, EPDS, and GDS, as well as indicators based on EHR documentation, diagnosis codes, and antidepressant medication use. Overall, ICD codes and PHQ-9 were the most consistently applied definitions across studies.

## DISCUSSION

EHR networks hold vast real-world data, yet their use in depression research is limited by multiple barriers. First, networks often provide complex and restrictive data access procedures. For example, EPIC Cosmos network requires skilled and certified Structured Query Language (SQL) engineers to access the data, who might not be readily available. Other networks, such as AllofUs and TriNetX, focus on reduction of entry barriers, but still might be difficult to navigate without specialized training. Educational programs, such as AIM AHEAD, are currently trying to address this labor gap.^86^ Other networks, such as PCORnet, require collaboration with the network’s data engineers to extract data, following a data request protocol, so the time to receive the data can be considerable and getting additional data ad-hoc beyond what is originally requested, might extend this time even more.

Second consideration is potential costs of using the networks, frequently exceeding thousands of dollars, present another barrier. More accessible networks like All of Us often provide limited datasets – only a few hundred thousand patients with complete data – lacking national representativeness, although other researchers noted that should not be a discouraging factor with appropriate study designs^119^. Epic Cosmos allows free access to data, but publications resulting from funded projects most likely require substantial payments, potentially limiting the pool of interested researchers.

Studies reviewed also mention additional access restriction imposed by EHR networks, such as: approval by oversight committees (e.g., NHS CHECK, QResearch, N3C Data Enclave, CRIS Oversight Committee); data use agreements, ethics training, or institutional collaboration (e.g., All of Us Researcher Workbench, iPSYCH, ENIGMA); licenses (CPRD). Some datasets in the networks are not open to unaffiliated researchers as mentioned in reviewed : “The datasets used and generated in this study are not openly available due to data confidentiality”^120^, or “Access to data is restricted to honorary or substantive employees of the South London and Maudsley NHS Foundation Trust and governed by a local oversight committee who review and approve applications to extract and analyse data for research”^121^, to give a few examples.

Most studies either do not mention how other researchers can access data or provide vague statements like “data available upon request” or “contact the corresponding author.”. Some reviewed studies explicitly state data is not publicly available or is restricted to approved collaborators (e.g., MBSC, KMBARC). Others mention partial availability, like genome-wide summary statistics on dbGaP or preliminary data via All of Us public data browser.

Other limitations of Real-World Data Sources which might present barriers to their use include problems with data quality^122^, such as EHR data can be incomplete or inconsistent, with missing values or errors in coding. Despite their large scale, many EHR research studies are concentrated in high-resource settings, and results may not generalize to underrepresented populations or global health systems without further validation.^8^ EHRs often lack information about care occurring outside the health system, and longitudinal care trajectories are susceptible to missingness when patients receive care elsewhere.^7^ In addition, depression diagnosis varies across providers and settings, leading to inconsistencies in how it is recorded in EHRs (e.g., differences in PHQ-9 vs. ICD-10 use). This variability complicates efforts to standardize depression phenotypes and generalize research findings. Researchers have raised concerns related to using patient data, which even when de-identified, raises privacy and ethical issues^123^. Strict regulations (e.g., HIPAA, GDPR) and data use agreements restrict access and sharing, as seen with networks like CPRD and All of Us.

## Conclusion

Data access challenges and data analysis complexity requires proficiency in specialized languages like R and Python are creating barriers for researchers without advanced technical skills. Potential biases, such as non-representative populations, risk skewed results and reduced generalizability, deterring use. Unfamiliarity with EHR networks and reliance on familiar datasets and methods further contribute to underutilization.

## Supplementary Material

Supplementary Files

This is a list of supplementary files associated with this preprint. Click to download.

- SupplementaryTablesS1S4.docx
- SupplementalFileS5.CompleteExtractedData.xlsx

## Figures and Tables

**Table 1:** Search strategy

**PubMed, Scopus, EBSCOHost**	(“All Of US” OR “AllofUs” OR “ENACT” OR “Million Veterans” OR “MVP” OR “TriNetX” OR “records research network” OR “EHR research network” OR “EMR research network” OR “PCORnet” OR “Cosmos” OR “Epic Cosmos” OR “ODSI” OR “Accrual To Clinical Trials” OR “National Clinical Cohort Collaborative” OR “N3C” OR “Datavant Switchboard” OR “Cerner Real World Data” OR “National COVID Cohort Collaborative” OR “Optum EHR” OR “Truveta”) AND depress*
Google Scholar	(EHR OR “health records”) “research network” “depression”

**Table 2:** Summary table of reviewed Research networks

Network name	References	Number of studies	Publication Years
TriNetX US Collaborative Network	7,9–41	34	2021–2024
All of Us Research Program	42–65	24	2022–2024
Million Veteran Program (MVP)	66–81	16	2020–2024
Veterans Health Administration (VHA)	see Supplemental File S5	9	2005–2023
Clinical Practice Research Datalink (CPRD)	“	5	2020–2024
QResearch	“	4	2018–2024
Mental Health Research Network (MHRN)	“	3	2016–2024
National COVID Cohort Collaborative (N3C)	“	3	2022–2023
PCORnetl Patient-Centered Clinical Research Network	“	3	2015–2023
Cerner Real-World Data	“	2	2023–2024
Environmental influences on Child Health Outcomes (ECHO)	“	2	2021–2023
Observational Health Data Sciences and Informatics (OHDSI)	“	2	2016–2021
OpenSAFELY	“	2	2023–2024
TRACK-TBI	“	2	2019–2021
